# Acupuncture for Anxiety in Lactating Mothers with Preterm Infants: A Randomized Controlled Trial

**DOI:** 10.1155/2013/169184

**Published:** 2013-11-24

**Authors:** Mariana Haddad-Rodrigues, Ana Márcia Spanó Nakano, Juliana Stefanello, Renata Cristina Campos Pereira Silveira

**Affiliations:** ^1^Ribeirão Preto School of Nursing, University of São Paulo, Ribeirão Preto, Rua José Lyra Filho, 110, apto 23A, 13309-340 Itu, SP, Brazil; ^2^Postgraduate Program in Public Health, Ribeirão Preto School of Nursing, University of São Paulo, Ribeirão Preto, SP, Brazil

## Abstract

The purpose of this study was to evaluate the effect of acupuncture versus placebo acupuncture on anxiety in lactating mothers with preterm infants. A parallel, randomized, patient-assessor blind, placebo-controlled trial was conducted in a tertiary school hospital in Londrina, Brazil, between 2011 and 2012. Mothers (*n* = 29) with very low birth weight infants born at this institution were randomly assigned to two treatment groups: acupuncture, AG (*n* = 14), or placebo acupuncture, PG (*n* = 15). Treatment sessions occurred once a week, using 5 Chinese auricular points unilaterally. The primary outcome measure was STAI-State scores, and secondary outcome measure was salivary cortisol levels. Both measures were collected before and after treatment and submitted to a blind assessor. Before-after treatment mean difference in STAI-State scores was observed in both groups (AG = 8.71 and PG = 8.20), not statistically significant (*P* = 0.888), although within group analysis was significant for both groups (*P* < 0.005). Salivary cortisol levels did not change after treatment in both groups (*P* = 0.480). There was no correlation between STAI and salivary cortisol results. At infant's hospital discharge, 76% subjects were breastfeeding exclusively. There was no difference between real and placebo acupuncture for anxiety in mothers with preterm infants.

## 1. Introduction

In the world, 20 million low birth weight infants are born each year. These children are 20 times more likely to die compared to babies born full-term, as they are more susceptible to several health problems [1]. In Brazil, in 2011, 35,000 children were born with very low birth weight (<1500 g) and 39% died under one year old [2].

An important way to prevent those deaths is breastfeeding. It is nutritive, cheap, and ecofriendly and improves both mother and child physical and emotional health. The World Health Organization (WHO) and the American Academy of Pediatrics recommend the use of human milk to nurture preterm infants [3].

Unfortunately there are some complications regarding the breastfeeding of very low birth weight (VLBW) infants. They often do not present the sucking-swallowing reflex required for successful breastfeeding. Their critical health condition calls for specialized hospital care in a neonatal intensive care unit (NICU) for a long period of time, usually spent inside an incubator. Also, they are submitted to many therapy essential interventions such as intravenous lines, respiratory and gastric tubes, and monitoring devices, which involve as little handling as possible. The separation from the child and the fear for its life may affect the mother's emotional status leading to many negative feelings, for instance, anxiety [4, 5].

The secretion of hormones involved with milk production (prolactin and oxytocin) is negatively influenced by the mother's anxiety, which hinders the onset and continuation of lactation [5, 6]. In order to stimulate milk production, health professionals recommend different approaches with positive results such as frequent milk expression, kangaroo-care, relaxation techniques, and use of galactogogue drugs [4].

Regarding the advantages of maternal milk to this feeble population of preterm babies and considering that improvement of mothers' emotional status could minimize this troublesome experience, the purpose of this study was to evaluate the effect of acupuncture versus placebo acupuncture on anxiety in lactating mothers with VLWB infants.

## 2. Acupuncture: Background and Challenges

Acupuncture was originated in China over 4,000 years ago and soon became popular in Japan and both North and South Korea, and shortly this technique was being used all over Asia. It was unknown to Europeans until the 17th century, and later, at the 1970s it reached the western part of the world. Since then, its efficacy has been questioned over the years [7], even though more and more people have come to know and use it.

Acupuncture has features that favor its use: it is safe and easy to apply. Contrary to many allopathic drugs, it is not toxic, it is not subjected to addiction or abuse, its side effects are scarce and minimum, and its contraindications are almost none. Besides, it is a simple and low-cost procedure and does not involve the use of expensive and high technology equipment [7].

Anxiety responses may be modulated with acupuncture through the endogenous opioid system [8], given that needle stimulation on acupoints prompts the release of 4 endo-opioids: enkephalin, *β*-endorphin, endomorphin, and dynorphin [9]. The effects of acupuncture in anxiety have been widely investigated and established in the scientific literature. This health condition among other mental health disorders, such as depression and insomnia, is the most common symptom reported by people who seek complementary treatments [10].

Regarding breastfeeding problems and hypogalactia, the use of acupuncture has specifically targeted milk production, using acupoints indicated for that complication. No study was found administering acupuncture for anxiety in order to aim mothers of preterm infants during the lactating and breastfeeding period, which was elected as object of this study.

## 3. Methods

This was a balanced randomization (1 : 1), patient-assessor blind, placebo-controlled, parallel-group study conducted at a tertiary school hospital in Londrina, Brazil.

### 3.1. Participants and Settings

Eligible participants were all women who gave birth to very low birth weight infants (<1500 g) who met the inclusion criteria as follows: (1) women's hospital discharge date within 7 days after delivery, (2) reading and writing literacy, (3) infant who was not breastfeeding, and (4) residence within 50 Km range from the hospital. Exclusion criteria were (1) use of galactogogues, (2) use of contraceptive pills or any other drug contraindicated during breastfeeding, (3) seropositivity for HIV or HTLV-1 and HTLV-2, (4) presenting with any health condition which contraindicated breastfeeding such as alcohol or drug abuse and psychiatric disorders, and (5) former acupuncture patients.

These selection criteria were designed considering that the milk expressed from the mother was to be given to her child, so the milk should meet specifications by the National Sanitation Agency [11], which regulates health, food, and sanitation procedures and products in Brazil. Also, volume of milk produced by mothers was considered as a secondary outcome measure, and if breastfeeding was initiated, it would bias data collection due to incapacity to measure milk ingested by the infant and the positive effect of sucking in milk production. However, the data collected was not reliable enough to be considered in the analysis; hence it is not mentioned or analyzed in this study. Furthermore, as placebo acupuncture was used to mimic real acupuncture, if the patient had former contact with this technique, they would be biased about this aspect. Subjects who lived in other cities too far away (>50 Km) from the hospital would have difficulty to visit the infant often and to comply with procedures included in the study design.

The school hospital where this study was conducted is the referral hospital for high risk pregnancies for Londrina and 20 other cities in the surrounding area. All health assistance is free-of-charge and supported by the Brazilian Ministry of Health. In 2012, the incidence of premature births was 39%, whereas the VLBW infants' birth rate was 9% [12]. 

### 3.2. Intervention Procedures

**Figure 1:**
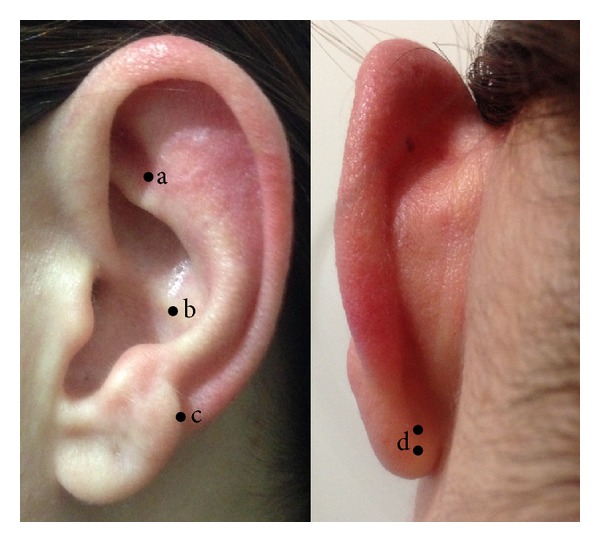
Location of ear acupoints. (a) shenmen; (b) muscle relaxation; (c) tension; (d) anxiety 1 and 2.

**Figure 2:**
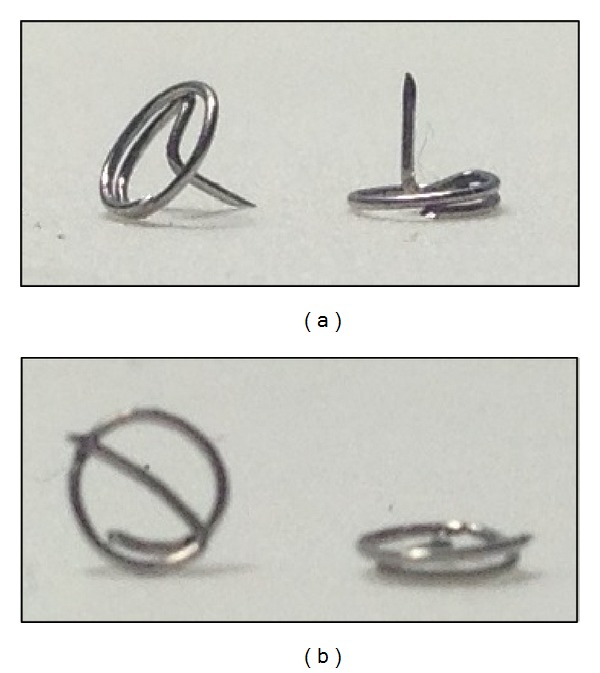
Needles used during intervention. (a) Real acupuncture needles; (b) placebo custom-made needles.

Patients were randomly assigned to one of two groups: real acupuncture (AG) or placebo acupuncture (PG). Treatment sessions occurred weekly, at the facilities of the institution's Human Milk Bank, where the participant remained seated during the 5-minute procedure. A total of 5 ear acupoints were selected based on their indication for anxiety, *Shenmen, tension, muscle relaxation, anxiety 1 and 2 *(Figure 1), and were located according to the Chinese Ear Acupuncture chart [13]. All 5 points were used in the sessions and retained until the next appointment. Initially the needles were inserted at subject's dominant side, applied in one ear at a time, alternating sides between sessions. Before application, the ear was sanitized with 70% alcohol and benzoin tincture was applied at acupoints to improve needle fixation. Real acupuncture was applied using sterile disposable stainless steel needles (1.0 mm × 1.5 mm), whilst placebo acupuncture was applied using the same needles customized to not perforate skin (Figure 2). A toothpick was used to create the sensation of needle perforation [14]. After needle insertion a 1 cm^2^beige micropore tape was placed on top of each needle for fixation. All procedures were provided by a licensed nurse acupuncturist with 5-year experience. 

The STAI-Trait and STAI-State scales were administered to subjects before intervention by a blinded independent data collector. They are 20-item self-answering questionnaires to measure trait anxiety, which is how a person responds to life stressors in general as a permanent feature inherent to them, and state anxiety, which is how a person responds to a life stressor they are experiencing at the present moment. The scores range from 20 to 80, directly proportional to the anxiety levels [15]. Only the STAI-state questionnaire was applied after the intervention.

Salivary cortisol levels were measured both before and after intervention at 2 time points: at day 0 and day 1 after the subject's hospital discharge and at day 0 and day 1 of infant's breastfeeding initiation. Two measures were taken before and after the intervention to strengthen the validity of variable and prevent that, in case of insufficient sample or loss, the other data should be accounted for. Subjects were given instructions and adequate material (*Salivette*, thermic box, and ice pack for sample transportation) to do the test at home, always at 11 pm. Subjects brought sample to the hospital, and then it was taken to a private laboratory for radioimmunoassay analysis.

The primary outcome was STAI-State scores before and after treatment, whereas salivary cortisol levels were considered as secondary outcome. Based on the primary outcome dependent variable, sample size was calculated considering high magnitude of treatment effect, *d* = 1.0 [16], with a two-sided 5% significance level and a power of 80%, and considering a 15% anticipated dropout rate, 20 subjects were needed per group.

### 3.3. Randomization, Blinding, and Data Analysis

Randomization sequence was created by a computer-generated list, using random block sizes of 4 and 6, with a 1 : 1 allocation. Sequential numbers were written outside opaque envelopes which contained the type of intervention inside. Each number corresponded to a participant, and all envelopes were prepared and sealed by another person so the researcher was blind to randomization. Participants were allocated after consent to take part in the study.

Participants were blind to which intervention they were receiving. Subjects from both groups shared the same hospital facilities, such as the NICU and Human Milk Bank, and could also identify themselves from other mothers who did not take part in the study. During the research participants never questioned the researcher about which group they were allocated to, and it seems that they did not compare perceptions regarding differences in intervention among themselves. The study design did not allow for researcher/acupuncturist blinding; however, the data analyst was blind to group allocation.

Data was analyzed using the Statistical Package for Social Sciences (SPSS) v.13.0 for Windows and G*Power v.3.03. STAI-State scores and salivary cortisol levels before and after treatment were compared using Student's *t*-test. Correlation between STAI and salivary cortisol results was assessed by Pearson's correlation. The significance level of 5% was assumed for all tests.

The study was approved by the institutional review board (Londrina School Hospital Research Ethics Committee) and registered in the Australian New Zealand Clinical Trials Registry (ANZCTR number 12611000025932). Informed consent was obtained voluntarily from all participants who received orientation about study goals and procedures.

## 4. Results

**Figure 3:**
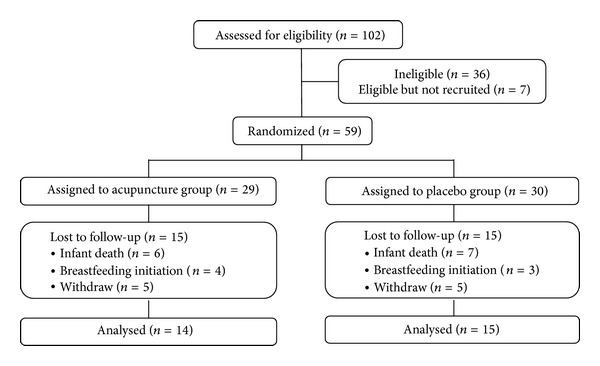
Flow diagram of a randomized, patient-assessor blind, placebo-controlled trial using acupuncture for anxiety in lactating mothers with premature infants.

This study was conducted from August 2011 to November 2012. Participants' enrollment, randomization, treatment allocation, follow-up, and analysis are described in Figure 3.

Considering all mothers with VLBW infants born at that institution, 58% were randomized. From this group only 49% completed the study protocol and had their data included in the analysis. Breastfeeding initiation before mother attended to at least 2 treatment sessions was an exclusion criteria after randomization. This minimal period was considered important for milk production initiation, considering volume of milk production as secondary outcome but later was excluded from analysis.

The sociodemographic and obstetric characteristics of subjects who completed the study protocol are displayed in Table 1. Homogeneity was verified between the groups for all variables, except for pregnancy planning (*P* = 0.031).

Participants' age ranged from 16 to 40, and mean age was 26, 86 years old. Most subjects had high school educational levels (57%), lived with their partners (72%), and were employed (65%). Cesarean birth was more prevalent (72%) which is expected at high risk pregnancies.

Mothers' breastfeeding and neonatal characteristics are shown in Table 2.

Among all participants, 48% had previous experiences with breastfeeding and 13% mentioned some problems while doing so. Exclusive breastfeeding rate at infant's hospital discharge was 76%, AG = 9 (64%), PG = 13 (87%), not significant (*P* = 0.215). Possibly this rate was higher in the PG because 46% of participants had previous breastfeeding experiences compared to 29% in the AG. Newborns' hospitalization period differed greatly among this sample, ranging from 29 to 174 days.

To measure the effect of acupuncture for anxiety in mothers with VLBW infants, STAI scale was applied to assess psychometric levels of anxiety (primary outcome), and salivary cortisol was used to biochemically quantify this variable (secondary outcome).

Participants' STAI scores before and after the intervention are mentioned in Table 3.

STAI-Trait was applied before intervention to establish a person's individual tendency to realize a given situation as stressful and to increase one's anxiety state while facing this situation. STAI-Trait scores ranged from 29 to 69, and both groups presented similar mean scores (AG = 44.57 and PG = 44.27), not statistically significant (*P* = 0.937).

STAI-State scores are able to identify subjective feelings such as tension, apprehension, and nervousness, which prompt an autonomic nervous system response [15]. STAI-S scores before intervention ranged from 22 to 66, and when mean scores were compared between groups, in the PG it was higher (50.07 to 46.43), not significant (*P* = 0.350). After intervention, mean scores dropped to 41.87 in the AG and to 37.71 in the PG. Although the score was lower for the PG participants, both groups presented similar mean differences comparing before-after intervention anxiety levels (AG = 8.71 and PG =8.20), not statistically significant between groups (*P* = 0.888). Conversely, within group analysis was statistically significant for both groups (*P* = 0.005). Magnitude of treatment effect revealed null or almost no effect (*d* = 0.05, IC = −5.08–4.95), and no significance was found.

Despite the fact that salivary cortisol testing has been used in several clinical studies to evaluate anxiety levels, it was not considered as the gold standard in this study. Concentrations of cortisol vary due to circadian cycle, and this may result in measurement instability [17]. Nocturnal salivary cortisol was measured based on the fact that cortisol levels are expected to be lower at this time of the day; therefore minimal changes could be detected. This procedure is applied to diagnose Cushing syndrome patients [18].

Table 4 contains participants' salivary cortisol results.

Preintervention cortisol measures were tested for homogeneity, and no statistical significance was found (*P* < 0.005). Before treatment, participants mean salivary cortisol concentrations were 0.13 ug/dL for both groups. The reference range for cortisol concentration in the saliva after 11 pm is <35 ug/dL. Postintervention measures ranged from 0.02 to 0.49 ug/dL, and 3 participants presented concentrations above reference range. Comparing pre- and postintervention values, salivary cortisol mean differences were −0.030 ug/dL in AG and −0.004 ug/dL in PG, not significant (*P* = 0.480).

Before and after treatment results from STAI scores and salivary cortisol values were assessed for correlation (Table 4). No correlation was found, except for STAI-State and salivary cortisol postintervention results in the PG (*P* = 0.007).

The protocol allowed for up to 12 weeks of treatment with the minimum of 2 sessions. Treatment duration ranged from 16 to 83 days, with mean of 41 days. Mean number of treatment sessions per subject was 5.55, with no statistical significance between groups (*P* = 0.463). The varied number of appointments could bias treatment results considering the cumulative effect of treatment. Pearson's correlation was applied to test number of sessions and STAI scores (*P* = 0.112) and salivary cortisol (*P* = 0.492) results, and no correlation was found.

## 5. Discussion

Most efficacy studies for anxiety using acupuncture were conducted in adults facing stressful situations such as dental extraction, performance anxiety, and elective surgery [10]. Conversely, many studies used acupuncture for hypogalactia or breast engorgement in women who gave birth to term infants and produced promising results [19–26]. Frequently, acupoints indicated for hypogalactia were applied, and the breastfeeding positive effect on milk production was not isolated as bias and mothers of preterm infants were not included in the sample. To our knowledge, there are no concluded studies that applied acupuncture for anxiety in mothers of preterm infants during the lactation period. 

Participant's STAI-S scores anxiety decreased, although no statistical significance was found between groups (Table 3). Hypothetically, if anxiety reduction was influenced by the treatment, we could imply that real whereas as placebo acupuncture produced the same effect on subjects' anxiety. Real acupuncture may have been as effective as the placebo effect caused by the participant's belief to be receiving acupuncture. Then, acupuncture was not effective compared to its placebo. Zhu et al. [27] analyzed different types of placebo acupuncture devices and concluded that all included needle-skin contact which could stimulate a sensory response. Even blunt needles, as the ones used in this study, could induce this reaction and originate its own therapeutic effect. Benedetti [28] explored 2 placebo-controlled clinical trials involving acupuncture for pain and found out that it had diminished in patients who assumed that they were allocated in the real acupuncture group, regardless of group allocation. Any intervention has a placebo component and the psychosocial is the major aspect of patient-care provider interaction and is contemplated as interpersonal treatment [28]. Future studies should consider the inclusion of a third control group with routine care for comparison between treatment groups and better establish the effects of each type of acupuncture (real or placebo).

Maybe anxiety decreased because mothers adapted to hospital routines and health situations concerning the infants' intensive care, eventually. Padovani et al. [29] studied a comparable population whose anxiety clinical symptoms decreased after infants' hospital discharge, and the same could be implied to this study's sample.

Contradicting STAI-state scores, salivary cortisol measures did not change after intervention and no statistical significance was found (*P* = 0.480) between groups (Table 4). Post-intervention measures were taken when infant's hospital discharge was being planned. The mother's new concerns and insecurities about how to take care of her baby, up to now under the care of the health team, may lead to increased anxiety. Feeley et al. [30] confirmed this premise among mothers at first week after infant's hospital discharge. 

Salivary cortisol measures were inconsistent regarding data of sample collection, storage, and transportation. These women suffered significant changes in lifestyle and routines and emotional status after the preterm birth which may have led to difficulty in taking the responsibility to do the test by themselves. Changes in meal and sleep schedules were common, and this is known to cause oscillation in cortisol concentrations. In this trial, this biologic anxiety indicator was not able to be controlled and modified by daily life activities and should be carefully considered whether it should be used in further studies targeting this population.

There was no correlation between STAI scores and salivary cortisol levels, except for post-intervention results in the PG (Table 5). Although statistically significant, when analyzed with other results altogether, this result should not be taken into account.

In conclusion, there was no difference between real and placebo acupuncture for anxiety in mothers with preterm infants. The study has some important limitations, such as lack of a third control group with routine care, small sample size, and elevated lost to follow-up. However, it was conducted and documented with strict methodological rigor. As such, it may raise new hypothesis for future trials on the effect of acupuncture and its benefits, exploring technique variations to find the most suitable for the population study, also providing contributions for clinical practice.

## Figures and Tables

**Table 1:** Sociodemographic and obstetric characteristics of subjects (*n* = 29).

Variable	AG (*n* = 14)		PG (*n* = 15)		Total		*P* value
Sociodemographic							
Age, mean (SD), min–max*, years	25,29 (6,51)	16–36*	28,33 (7,91)	16–40*	26,86 (7,30)	16–40*	0,269^a^
Race, *n* (%)							
Caucasian	7	(50)	7	(46,7)	14	(48,3)	1,000^b^
Noncaucasian	7	(50)	8	(53,3)	15	(51,7)	
Level of education, *n* (%)							
Elementary and middle school	5	(35,7)	2	(13,3)	7	(24,1)	0,949^c^
High school	5	(35,7)	12	(80,0)	17	(58,6)	
University degree	4	(28,6)	1	(6,7)	5	(17,2)	
Conjugal status, *n* (%)							
With partner	10	(71,4)	11	(73,3)	21	(72,4)	1,000^b^
Without partner	4	(28,6)	4	(26,7)	8	(27,6)	
Family income, *n* (%), minimum wage							
<1	4	(28,6)	2	(14,3)	6	(21,4)	0,769^c^
1–3	8	(57,1)	11	(67,9)	19	(67,9)	
>3	2	(14,3)	1	(7,1)	3	(10,7)	
Employed, *n* (%)							
Yes	8	(57,1)	11	(73,3)	19	(65,5)	0,450^b^
No	6	(42,9)	4	(26,7)	10	(34,5)	
Alcohol use, *n* (%)							
Yes	1	(7,1)	2	(13,3)	3	(10,3)	1,000^b^
No	13	(92,9)	13	(89,7)	26	(89,7)	
Tobacco use, *n* (%)							
Yes	2	(14,3)	0	(6,9)	2	(6,9)	0,224^b^
No	12	(85,7)	15	(93,1)	27	(93,1)	
Place of residence, *n* (%)							
Londrina	8	(57,1)	12	(80,0)	20	(69,0)	0,245^b^
Other cities	6	(42,9)	3	(20,0)	9	(31,0)	
Obstetric							
Parity, *n* (%)							
Primiparous	4	(28,60)	7	(46,70)	14	(48,30)	0,450^b^
Multiparous	10	(71,40)	8	(53,30)	15	(51,70)	
Type of birth, *n* (%)							
Vaginal	3	(21,4)	5	(33,3)	8	(27,6)	0,682^b^
Cesarean	11	(78,6)	10	(66,7)	21	(72,4)	
Pregnancy was planned, *n* (%)							
Yes	6	(42,9)	6	(40,0)	12	(41,4)	1,000^b^
No	8	(57,1)	9	(58,6)	17	(58,6)	
Prenatal appointments, mean (SD), min–max*	7,79 (3,29)	5–16*	5,33 (2,47)	2–10*	6,52 (3,10)	2–16*	0,031^a^
No. of pregnancies, mean (SD), min–max*	2,43 (2,31)	1–9*	1,93 (1,39)	1–5*	2,17 (1,87)	1–9*	
No. of vaginal births, mean (SD), min–max*	0,71 (0,99)	0–3*	0,73 (1,10)	0–4*	0,72 (1,03)	0–4*	
No. of cesareans, mean (SD), min–max*	1,00 (0,68)	0–3*	1,00 (0,85)	0–3*	1,00 (0,76)	0–3*	
Abortion, *n* (%)							
Yes	4	(26,6)	1	(6,7)	5	(17,2)	
No	10	(71,4)	14	(93,3)	24	(82,8)	

SD: standard deviation. ^a^Student's *t*-test; ^b^Fisher's exact test; ^c^Pearson's correlation test.

**Table 2:** Breastfeeding and neonatal characteristics.

Variable	AG (*n* = 14)		PG (*n* = 15)		Total		*P* value
Breastfeeding							
Previous experience, *n* (%)							
Yes	4	(28,60)	7	(46,70)	14	(48,30)	0,450*
No	10	(71,40)	8	(53,30)	15	(51,70)	
Breastfeeding problems, *n* (%)							
Yes	1	(7,10)	3	(20,00)	4	(13,80)	0,598*
No	13	(92,90)	12	(80,00)	25	(86,20)	
Galactogogue (protocol completed), *n* (%)							
Yes	6	(42,90)	6	(40,00)	14	(48,30)	1,000*
No	8	(57,10)	9	(60,00)	15	(51,70)	
Exclusively breastfeeding at infant's hospital discharge							
Yes	9	(64,30)	13	(86,70)	22	(75,90)	0,215*
No	5	35,70)	2	(13,30)	7	(24,10)	
Neonatal							
Gender, *n* (%)							
Female	9	(64,3)	8	(53,3)	17	(58,6)	
Male	5	(35,7)	7	(46,7)	12	(41,4)	
Size to gestational age, *n* (%)							
Adequate	9	(64,3)	12	(80,0)	21	(72,4)	
Small	5	(35,7)	3	(20,0)	8	(27,6)	
Birth weight, mean (SD), min–max*, grams	1046,79 (248,88)	535–1335*	1081,67 (229,98)	620–1440*	1064,83 (253,62)	535–1440*	
Gestational age, mean (SD), median* (min–max), weeks	29,06 (2,42)	24,86–34,00*	28,86 (2,76)	24,29–34,43*	28,96 (2,56)	24,29–34,43*	
1-minute Apgar score, mean (SD), min–max*	5,21 (2,19)	1–8*	5,07 (2,60)	1–9*	5,15 (2,37)	1–9*	
5-minute Apgar score mean (SD), min–max*	7,64 (1,39)	4–9*	8,07 (0,96)	7–10*	7,86 (1,19)	4–10*	
Hospitalization period, mean (SD), min–max*, days	64,43 (24,01)	34–119*	62,27 (35,28)	29–174*	63,31 (29,85)	29–174*	

SD: standard deviation.

*Student's *t*-test.

**Table 3:** STAI scores before and after the intervention.

STAI scores	Minimum	Maximum	Mean	Standard deviation	*P* value
STAI-Trait (preintervention)					
AG (*n* = 14)	29,00	69,00	44,57	11,64	0,937^a^
PG (*n* = 15)	29,00	61,00	44,27	8,71	
Total (*n* = 29)	29,00	69,00	44,41	10,05	
STAI-State (postintervention)					
AG	28,00	66,00	46,43	11,10	0,350^a^
PG	32,00	63,00	50,07	9,48	
Total	28,00	66,00	48,31	10,28	
STAI-State (postintervention)					
AG	28,00	50,00	37,71	5,33	
PG	31,00	56,00	41,87	8,85	
Total	28,00	56,00	39,86	7,53	
Difference (pre- and postintervention)*					
AG	−4,00	30,00	8,71	9,79	0,888^a^
PG	−6,00	24,00	8,20	9,68	
Total	−6,00	30,00	8,45	9,56	

*STAI-State preintervention minus STAI-State postintervention. ^a^Student's *t*-test

**Table 4:** Salivary cortisol levels before and after intervention (ug/dL).

Variables	Minimum	Maximum	Mean	Standard deviation	*P* value
Preintervention measures^1^					
AG (*n* = 14)	0,04	0,33	0,13	0,08
PG (*n* = 15)	0,02	0,31	0,13	0,08
Total (*n* = 29)	0,02	0,33	0,13	0,08	
Postintervention measures^2^					
AG	0,02	0,49	0,14	0,15
PG	0,02	0,35	0,14	0,09
Total	0,02	0,49	0,14	0,12	
Difference (pre- minus postintervention)^3^					
GA	−0,35	0,13	-0,03	0,14	0,480**
GP	−0,20	0,14	0,00*	0,09	
Total	−0,35	0,14	-0,02	0,11	

^1^Mean value of day 0 and day 1 measurements after subject's hospital discharge.

^2^Mean value of day 0 and day 1 measurements of infant's successful breastfeeding initiation.

^3^Preintervention measurements minus postintervention measurements.

*−0.004

**Student's *t*-test.

**Table 5:** Correlation between STAI scores and salivary cortisol levels (*P* value).

Variables	Preintervention cortisol			Postintervention cortisol		
	AG	PG	Total	AG	PG	Total
STAI-Trait	0,402	0,252	0,154	0,239	0,041	0,800
STAI-State (pre-intervention)	0,749	0,942	0,753	—	—	—
STAI-State (post-intervention)	—	—	—	0,676	0,007*	0,388

*Pearson's correlation (*P* < 0.05).
